# Lack of Association between Interleukin-10 Gene Polymorphisms and Graft Rejection Risk in Kidney Transplantation Recipients: A Meta-Analysis

**DOI:** 10.1371/journal.pone.0127540

**Published:** 2015-06-02

**Authors:** Jiachuan Xiong, Yiqin Wang, Ying Zhang, Ling Nie, Daihong Wang, Yunjian Huang, Bing Feng, Jingbo Zhang, Jinghong Zhao

**Affiliations:** Department of Nephrology, Institute of Nephrology of Chongqing and Kidney Center of PLA, Xinqiao Hospital, Third Military Medical University, Chongqing, 400037, China; Centre for Inflammation Research, UNITED KINGDOM

## Abstract

**Background:**

Interleukin-10 (IL-10) is an important immunomodulatory cytokine. Several studies focused the association between IL-10 promoter gene polymorphisms and graft rejection risk in kidney transplantation recipients. However, the results of these studies remain inconclusive. The aim of this study was to conduct a meta-analysis to further assess the associations.

**Methods:**

The PubMed, Embase, and Ovid Medline databases were searched. Two independent authors extracted data, and the effects were estimated from an odds ratio (OR) with 95% confidence intervals (CIs). Subgroup and sensitivity analyses identified sources of heterogeneity.

**Results:**

A total of 16 studies including 595 rejection patients and 1239 stable graft patients were included in order to study the IL-10 -1082 (rs1800896 G/A), -819 (rs1800871 C/T), -592 (rs1800872 C/A) and IL-10 (-1082,-819,-592) polymorphisms. The -1082 G/A polymorphism was not associated with an increased graft rejection risk (OR = 1.03; 95%CI, 0.85–1.25, P = 0.74 for GA+AA vs. GG model). Moreover, all of the -819 C/T (OR = 1.06, 95%CI, 0.79–1.42, P = 0.70 for TA+TT vs. CC model), -592 C/A (OR = 1.10, 95% CI, 0.85–1.42, P = 0.47 for AC+AA vs. CC model) and IL-10 (-1082,-819,-592) polymorphisms (OR = 1.00, 95%CI, 0.79–1.27, P = 0.98 for I+L vs. H model) did not increase the graft rejection risk. In addition, we also performed subgroup analysis by ethnic group (mainly in Europeans or Asians) and rejection type (acute or chronic). There was also lack of evidence of a significant association between the IL-10 gene polymorphism and graft rejection risk. The present meta-analysis indicated that the IL-10 gene polymorphism was not associated with graft rejection risk in kidney transplantation recipients.

**Conclusion:**

This meta-analysis found evidence that the IL-10 polymorphism does not increase the risk of graft rejection in kidney transplantation recipients. Further chronic rejection and other ethnic population studies are needed to confirm our results.

## Introduction

Worldwide, kidney transplantation (KTx) is recognized as a treatment for end-stage renal disease (ESRD) [1]. It provides a better quality and quantity of life than dialysis treatments for its cost effectiveness [2]. Patient survival for those who received a kidney transplant has progressively improved towards 90%, and 1-year graft survival for deceased donor kidney transplantation has increased to 91% in Europeans [3]. However, acute rejection episodes and delayed graft function after KTx remain a clinical concern [4]. Acute rejection may result in graft loss, increased risk of chronic allograft dysfunction, and poor long-term outcomes [5]. Delayed graft function also predisposes patients to worse long-term outcomes by increasing the risk for acute rejection and subsequent chronic allograft dysfunction [6]. Despite the currently advanced immunosuppressive therapy that can effectively decrease the rejection rate of KTx [7], the increased risk of cancer, nephrotoxicity, and infection and other drug-related side effects should be considered and need careful management [8, 9].

Previous studies show that patients’ genetic background was associated with allograft outcome [10], which can be mediated by immune dysregulation and inflammation infiltration. Cytokines are key mediators and play a critical role in the induction and effector phases of all immune and inflammatory responses [11]. Genetic polymorphisms of the cytokine genes are suggested to correlate with immunological activity after organ transplantation [12], particularly within the promoter regions of TNF-a, IL-10, IL-6, and TGF-b [13]. Polymorphisms in interleukin (IL)-10 have been among the most extensively analyzed, but the results of analyses to determine the association between IL-10 promoter gene polymorphisms and graft rejection risk have been inconclusive.

The aim of the present study was to determine whether analysis of IL-10 gene polymorphisms can be used to identify patients at increased risk for acute rejection or delayed graft function. We performed this meta-analysis to evaluate the association between IL-10 gene polymorphisms and graft rejection in recipients after kidney transplantation.

## Methods

### Data sources and searching term

We performed a search to identify the studies that examined the associations between Interleukin-10 gene polymorphisms and graft rejection risk in kidney transplantation recipients. Literature was identified by searching the Ovid Medline, Embase, and PubMed databases. The last updated search was performed in August 2014. The searching terms used were ‘‘Interleukin-10 gene or IL-10”, ‘‘polymorphism”, ‘‘rejection or graft failure”, and “renal transplantation or kidney transplantation or renal transplant”. The search was carried out without restriction on language but was limited to studies that had been conducted on human subjects.

### Inclusion criteria

The included studies met the following criteria: (1) evaluated IL-10 gene polymorphism and graft rejection risk (i.e., IL-10: -1082 (rs1800896 G/A), -819 (rs1800871 C/T), -592 (rs1800872 C/A), IL-10(-1082,-819,-592)); (2) for case–control studies, the clinical outcome were defined as acute rejection or chronic rejection; (3) supplied the number of individual genotypes in rejection cases and no rejection cases, having a stable graft function group as a comparator; and (4) indicated that the distributions of genotypes in cases and controls were available for estimating odds ratio (OR) with a 95% confidence interval (CI).

### Data extraction

Two authors independently extracted the information from all eligible publications using standard data extraction forms. Disagreements were resolved by discussion between the two authors or through consultation with a third reviewer. A standardized data form was used for data collection and included the following: first author, year of publication, country of origin, ethnicity of the study population, definition of rejection cases, no rejection cases, genotyping methods, and genotype distribution in cases and controls.

### Evaluation of publication bias

The publication bias was evaluated using Egger’s linear regression test, which measures funnel plot asymmetry using a natural logarithm scale of odds ratios (ORs), and subgroup analyses were also performed to determine the heterogeneities.

### Evaluations of statistical associations

We performed meta-analysis on (1) allelic contrast, (2) recessive, (3) dominant, and (4) additive models. And we use dominant model as a representative model to explain the results. Point estimates of risks, ORs, and 95% confidence intervals (CI) were estimated for each study. We also assessed within- and between-study variations and heterogeneities using Cochran’s Q-statistic. Heterogeneity was analyzed using a chi squared test on N-1 degrees of freedom, with an alpha of 0.05 used for statistical significance and with the I^2^ test. I^2^ values of 25%, 50% and 75% correspond to low, medium and high levels of heterogeneity, respectively. A random-effects model was used if the studies exhibited heterogeneity (I^2^ values >30%); otherwise, the fixed-effects model was selected. To evaluate the ethnicity-specific effects, subgroup analysis was performed by ethnicity and by acute rejection or delayed graft dysfunction. The Begg’s test and the Egger’s test were used to assess publication bias statistically. Hardy-Weinberg equilibrium (HWE) was tested with the Pearson’s v^2^ test. STATA software (version 12.0) was used to perform all statistical analyses.

## Results

### Study characteristics

**Fig 1:**
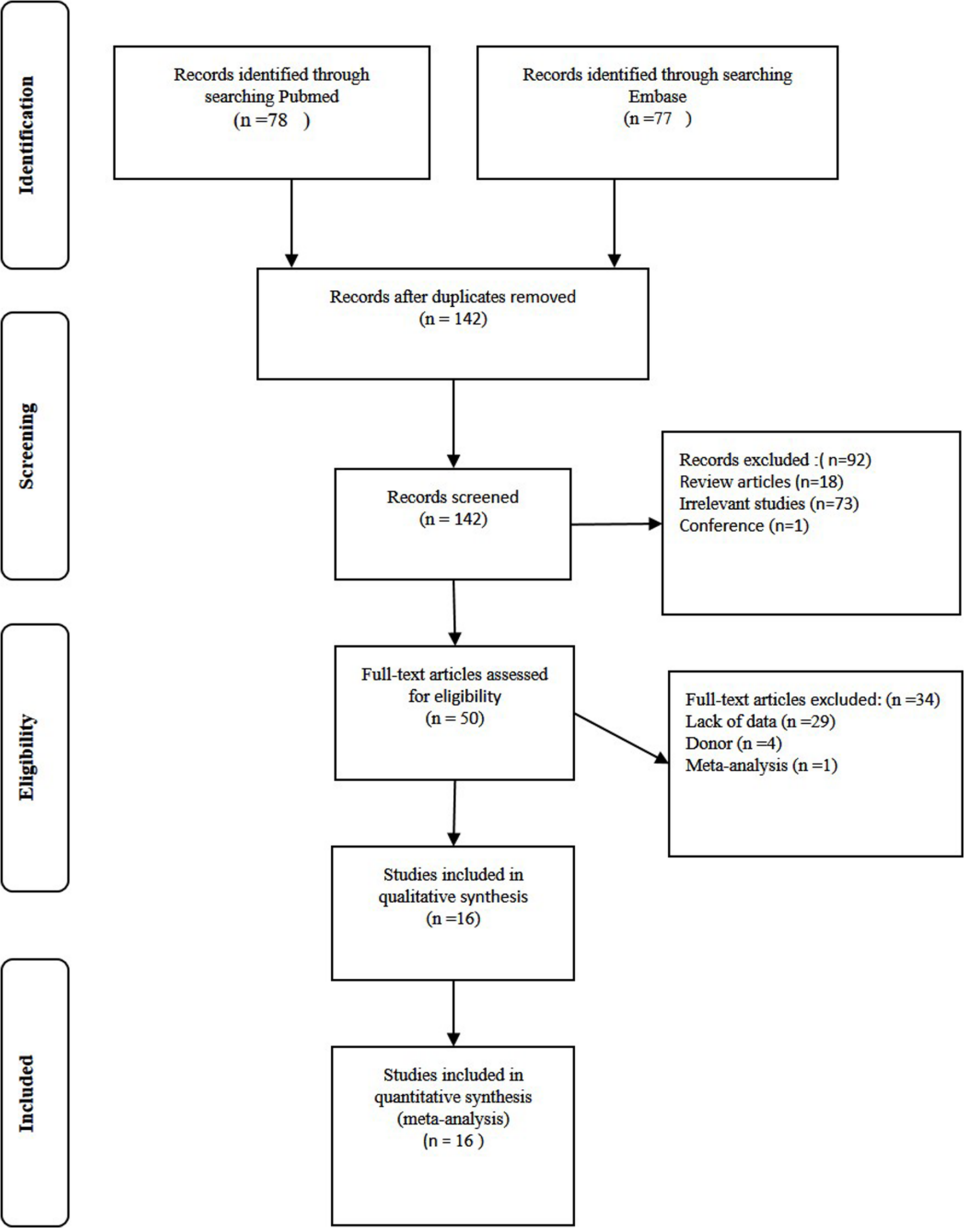
Flow diagram of included/excluded studies.

The flow diagram for the literature search is shown in Fig 1. The primary screening identified 155 potentially relevant articles, and 139 studies were excluded after reading the title or abstract because of obvious irrelevance to our study aim and lack of data for calculation. Finally, a total of 16 studies including 595 rejection patients and 1239 stable graft patients met the criteria [14–21, 4, 22–28]. Seven articles were from European population, 6 studies were from Asian populations, and one study each was performed with an African and mixed population. Eleven studies were performed to evaluate the IL-10-1082 G/A polymorphism, while there were six studies that evaluated the association between the IL-10-819 C/T, IL-10-592 C/A and IL-10-(-1082,-819,-592) gene polymorphisms and graft rejection risk. The basic characteristics of the included studies are listed in Table 1.

**Table 1:** Characteristics of the studies included in the meta-analysis.

Author	Year	Country	Ethnicity	Reject/stable graft function	Genotype method	Polymorphism
**Marshall** ^**14**^	2000	United Kingdom	European	114/95	PCR-SSP	-1082 G/A,-819 C/T,-592 C/A,IL-10 (-1082,-819,-592)
**George** ^**15**^	2001	United Kingdom	European	34/71	PCR	-1082 G/A
**Melk** ^**16**^	2003	Germany	European	25/14	RT-PCR	-1082 G/A,-819 C/T,-592 C/A
**McDaniel** ^**17**^	2003	USA	American	29/41	RT-PCR	IL-10 (-1082,-819,-592)
**Dmitrienko** ^**18**^	2005	Canada	European	50/50	PCR-SSP	-1082 G/A
**Gendzekhadze** ^**19**^	2006	Venezuela	Mix	30/33	PCR-SSP	-1082 G/A,-819 C/T,-592 C/A
**Tajik** ^**20**^	2006	Iran	Asian	11/31	PCR-SSP	IL-10 (-1082,-819,-592)
**Canossi** ^**21**^	2007	Italy	European	25/61	PCR-SSP	IL-10 (-1082,-819,-592)
**Amirzargar** ^**4**^	2007	Iran	Asian	22/78	PCR-SSP	-1082 G/A,-819 C/T,-592 C/A
**Manchanda** ^**22**^	2008	India	Asian	18/82	PCR-RFLP	-1082 G/A,-819 C/T
**Azarpira** ^**23**^	2009	Iran	Asian	46/54	PCR-ARMS	-1082 G/A
**Omrani** ^**24**^	2010	Iran	Asian	32/52	PCR	-592 C/A
**Seyhun** ^**25**^	2012	Turkey	European	19/71	PCR-SSP	-1082 G/A,-819 C/T,-592 C/A,IL-10 (-1082,-819,-592)
**Dhaouadi** ^**26**^	2013	Tunisia	African	80/151	PCR-SSP	IL-10 (-1082,-819,-592)
**Chang** ^**27**^	2013	Taiwan	Asian	43/280	PCR-RFLP	-592 C/A
**Gaafar** ^**28**^	2014	Saudi Arabia	Asian	17/75	PCR-SSP	-592 C/A

Note: Mix means allograft recipients donors were mestizos; PCR-SSP: Polymerase Chain Reaction-Sequence Specific Primer; PCR: Polymerase Chain Reaction; RT-PCR: Real-time polymerase chain reaction; PCR-RFLP: Polymerase Chain Reaction-Restriction Fragment Length Polymorphism; PCR-ARMS: Polymerase Chain Reaction-Amplification Refractory Mutation System.

### Meta-analysis of the -1082 G/A polymorphism and graft rejection risk

**Fig 2:**
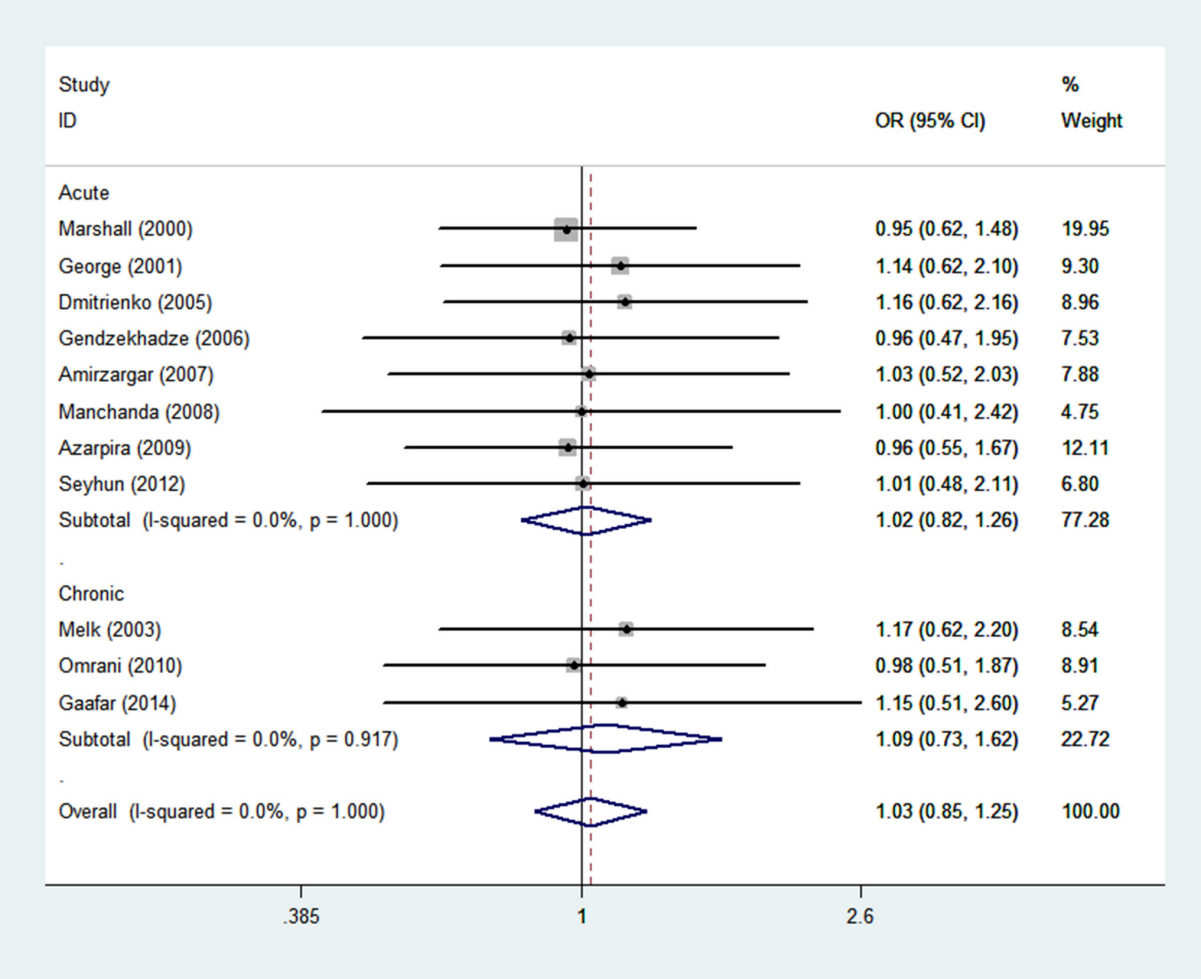
Meta-analysis with a fixed effect model for the association between reject risk and the -1082 G/A polymorphism (GA+AA VS GG).

There were a total of 407 rejection cases and 675 controls in 11 case-control studies that were included in the meta-analysis on the relationship between the -1082 G/A polymorphism and the risk of graft rejection. The study numbers for acute rejection and chronic graft rejection were 8 and 3, respectively. Five studies were from a European population, 5 were from an Asian population, and 1 was from mix ethnic population. We found that there was no association between the -1082 G/A polymorphism and graft rejection risk (OR = 1.03; 95% CI, 0.85–1.25, P = 0.74 for GA+AA vs. GG model, Fig 2 and Table 2). For AA vs. GA+GG model, the -1082 G/A polymorphism was associated and graft rejection risk (OR = 1.34; 95% CI, 1.02–1.75, P = 0.04). By subgroup analysis, we did not find that the -1082 G/A polymorphism increased the risk of acute rejection (OR = 1.02; 95%CI, 0.82–1.27, P = 0.88, Fig 2 and Table 2) or chronic rejection (OR = 1.09; 95%CI, 0.73–1.62, P = 0.74, Fig 2 and Table 2). Moreover, in the subgroup meta-analysis by ethnicity, we found similar results that the -1082 G/A polymorphism did not significantly increase risks among European (OR = 1.06, 95%CI, 0.82–1.38, P = 0.65, S1 Fig in supplementary and Table 2) or Asian populations” (OR = 1.01, 95%CI, 0.74–1.37, P = 0.97, S1 Fig and Table 2). But in the one study of other ethic, for AA vs. GA+GG model, the -1082 G/A polymorphism was associated and graft rejection risk (OR = 1.22; 95% CI, 0.55–2.74, P = 0.04). A summary of the results of other genetic comparisons is shown in Table 2.

**Table 2:** Meta-analysis of associations between the IL-10 gene polymorphisms and rejection in kidney transplantation.

Position	No.	Sample size	AG VS GG		AA VS GG		GA+AA VS GG		AA VS GA+GG		A VS G	
**-1082 G/A**			OR(95% CI)	P	OR(95% CI)	P	OR(95% CI)	P	OR(95% CI)	P	OR(95% CI)	P
**total**	11	407/675	0.99[0.78,1.26]	1.00	1.20[0.89,1.62]	0.23	1.03[0.85,1.25]	0.74	1.34[1.02,1.75]	0.04	1.11[0.95,1.29]	0.19
**Acute**	8	333/534	0.96[0.73,1.26]	1.00	1.16[0.84,1.62]	0.37	1.02[0.82,1.27]	0.88	1.33[0.98,1.80]	0.07	1.09[0.92,1.30]	0.31
**Chronic**	3	74/141	1.09[0.68,1.75]	0.83	1.36[0.70,2.64]	0.37	1.09[0.73,1.62]	0.74	1.34[1.02,1.75]	0.31	1.15[0.83,1.57]	0.40
**European**	5	242/301	1.03[0.74,1.43]	0.87	1.27[0.86,1.86]	0.23	1.06[0.82,1.38]	0.65	1.33[0.93,1.89]	0.12	1.13[0.92,1.39]	0.24
**Asian**	5	135/341	0.96[0.67,1.38]	0.87	1.17[0.67,2.04]	0.57	1.01[0.74,1.37]	0.97	1.40[0.86,2.28]	0.18	1.08[0.84,1.38]	0.55
**others**	1	30/33	0.86[0.26,2.79]	0.80	0.96[0.40,2.32]	0.93	0.96[0.47,1.95]	0.92	1.22[0.55,2.74]	0.04	1.06[0.62,1.79]	0.84
**-819 C/T**			TC VS CC		TT VS CC		TA+TT VS CC		TT VS TC+CC		T VS C	
**total**	6	228/373	1.05[0.76,1.46]	0.76	1.20[0.69,2.09]	0.51	1.06[0.79,1.42]	0.70	1.10[0.66,1.86]	0.71	1.07[0.84,1.37]	0.60
**Acute**	5	203/359	0.99[0.69,1.41]	0.96	1.16[0.63,2.16]	0.63	1.01[0.74,1.39]	0.94	1.10[0.62,1.97]	0.74	1.03[0.79,1.35]	0.83
**Chronic**	1	25/14	1.49[0.63,3.54]	0.37	1.38[0.39,4.84]	0.62	1.35[0.64,2.87]	0.43	1.10[0.33,3.73]	0.88	1.29[0.70,2.41]	0.42
**European**	3	158/180	1.03[0.68,1.57]	0.88	1.27[0.60,2.70]	0.54	1.04[0.71,1.52]	0.84	1.20[0.57,2.53]	0.62	1.07[0.77,1.48]	0.70
**Asian**	2	40/160	1.17[0.62,2.19]	0.64	1.53[0.60,3.90]	0.37	1.20[0.70,2.05]	0.52	1.26[0.55,2.89]	0.58	1.21[0.78,1.87]	0.39
**others**	1	30/33	0.93[0.37,2.33]	0.87	0.45[0.08,2.61]	0.37	0.87[0.38,2.01]	0.74	0.44[0.08,2.44]	0.35	0.78[0.38,1.59]	0.49
**-592 C/A**			AC VS CC		AA VS CC		AC+AA VS CC		AA VS AC+CC		A VS C	
**total**	6	253/571	1.13[0.83,1.53]	0.45	1.07[0.70,1.64]	0.75	1.10[0.85,1.42]	0.47	1.08[0.72,1.61]	0.71	1.10[0.89,1.35]	0.40
**Acute**	5	228/557	1.08[0.78,1.50]	0.65	1.04[0.66,1.63]	0.88	1.07[0.81,1.41]	0.64	1.08[0.71,1.65]	0.73	1.07[0.86,1.34]	0.55
**Chronic**	1	25/14	1.49[0.63,3.54]	0.37	1.38[0.39,4.84]	0.62	1.35[0.64,2.87]	0.43	1.10[0.33,3.73]	0.88	1.29[0.70,2.41]	0.42
**European**	3	158/180	1.24[0.83,1.84]	0.30	1.27[0.60,2.70]	0.54	1.20[0.83,1.73]	0.33	1.14[0.54,2.38]	0.74	1.10[0.87,1.63]	0.28
**Asian**	2	65/358	1.01[0.57,1.78]	0.99	1.08[0.62,1.86]	0.79	1.05[0.70,1.60]	0.83	1.15[0.70,1.90]	0.58	1.08[0.79,1.48]	0.63
**others**	1	30/33	0.93[0.37,2.33]	0.87	0.45[0.08,2.61]	0.37	0.87[0.38,2.01]	0.74	0.44[0.08,2.44]	0.35	0.78[0.38,1.59]	0.49
**IL(1082,819,592)**			I VS H		L VS H		I+L VS H		I VS L+H			
**total**	6	278/450	1.03[0.77,1.39]	0.84	0.96[0.67,1.36]	0.80	1.00[0.79,1.27]	0.98	0.94[0.69,1.27]	0.67	-	-
**Acute**	5	249/409	1.01[0.74,1.38]	0.96	0.90[0.62,1.31]	0.59	0.98[0.77,1.26]	0.89	0.84[0.61,1.18]	0.31	-	-
**Chronic**	1	29/41	1.31[0.49,3.50]	0.59	1.56[0.52,4.68]	0.43	1.21[0.57,2.55]	0.62	2.18[0.83,5.67]	0.11	-	-
**European**	3	158/227	1.07[0.71,1.61]	0.75	0.84[0.51,1.39]	0.50	0.99[0.71,1.39]	0.97	0.66[0.42,1.03]	0.07	-	-
**Asian**	1	11/31	0.76[0.21,2.70]	0.67	0.75[0.14,3.90]	0.73	0.84[0.29,2.38]	0.74	1.08[0.25,4.57]	0.92	-	-
**others**	2	109/192	1.03[0.65,1.64]	0.90	1.12[0.67,1.87]	0.67	1.04[0.73,1.48]	0.85	1.33[0.85,2.09]	0.21	-	-

### Meta-analysis of the -819 C/T polymorphism and graft rejection risk

**Fig 3:**
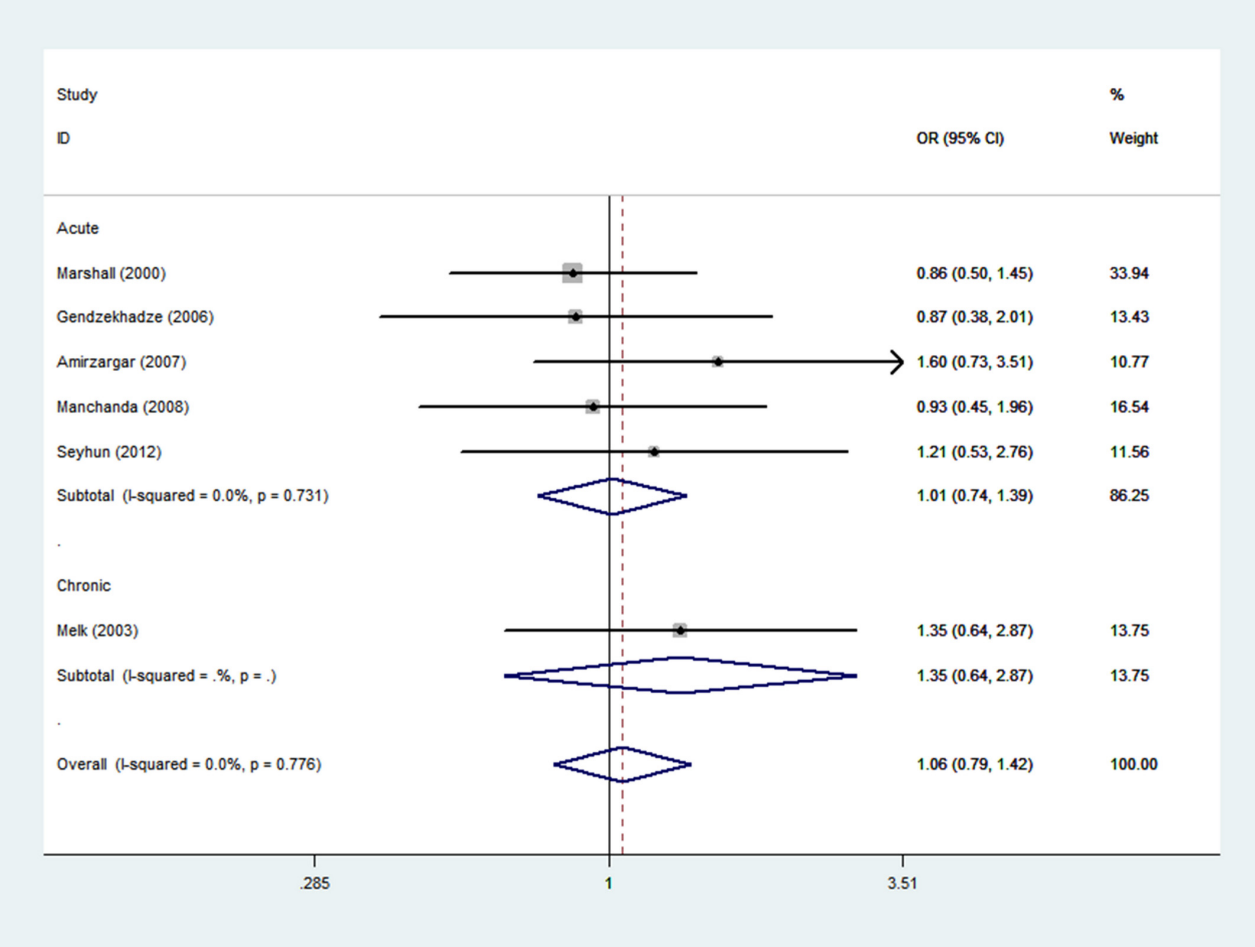
Meta-analysis with a fixed effect model for the association between reject risk and the -819 C/T polymorphism (TA+TT VS CC).

A total of 228 rejection cases and 373 controls in 6 case-control studies were included in the meta-analysis of the relationship between the -819 C/T polymorphism and the risk of graft rejection. There were 5 studies on acute rejection and 1 study on chronic graft rejection. Three studies were from European populations, 2 were from Asian populations, and 1 was mix ethnic population. Overall, there was no statistical evidence of an association between the -819 C/T polymorphism and overall graft rejection risks (OR = 1.06, 95%CI, 0.79–1.42, P = 0.70 for TA+TT vs. CC model, Fig 3 and Table 2). In the subgroup analysis by type of rejection, no significantly increased risk was found among acute rejection (OR = 1.01, 95%CI, 0.74–1.39, P = 0.94, Fig 3 and Table 2) and chronic rejection (OR = 1.35, 95%CI, 0.64–2.87, P = 0.43, Fig 3 and Table 2), and no significantly increased risk was found in either Europeans (OR = 1.04, 95% CI, 0.71–1.52, P = 0.84, S2 Fig and Table 2) or in Asians (OR = 1.20, 95%CI, 0.70–2.05, P = 0.52, S2 Fig and Table 2) in the ethnic analysis. A summary of the results of other genetic comparisons is shown in Table 2.

### Meta-analysis of the -592 C/A polymorphism and graft rejection risk

**Fig 4:**
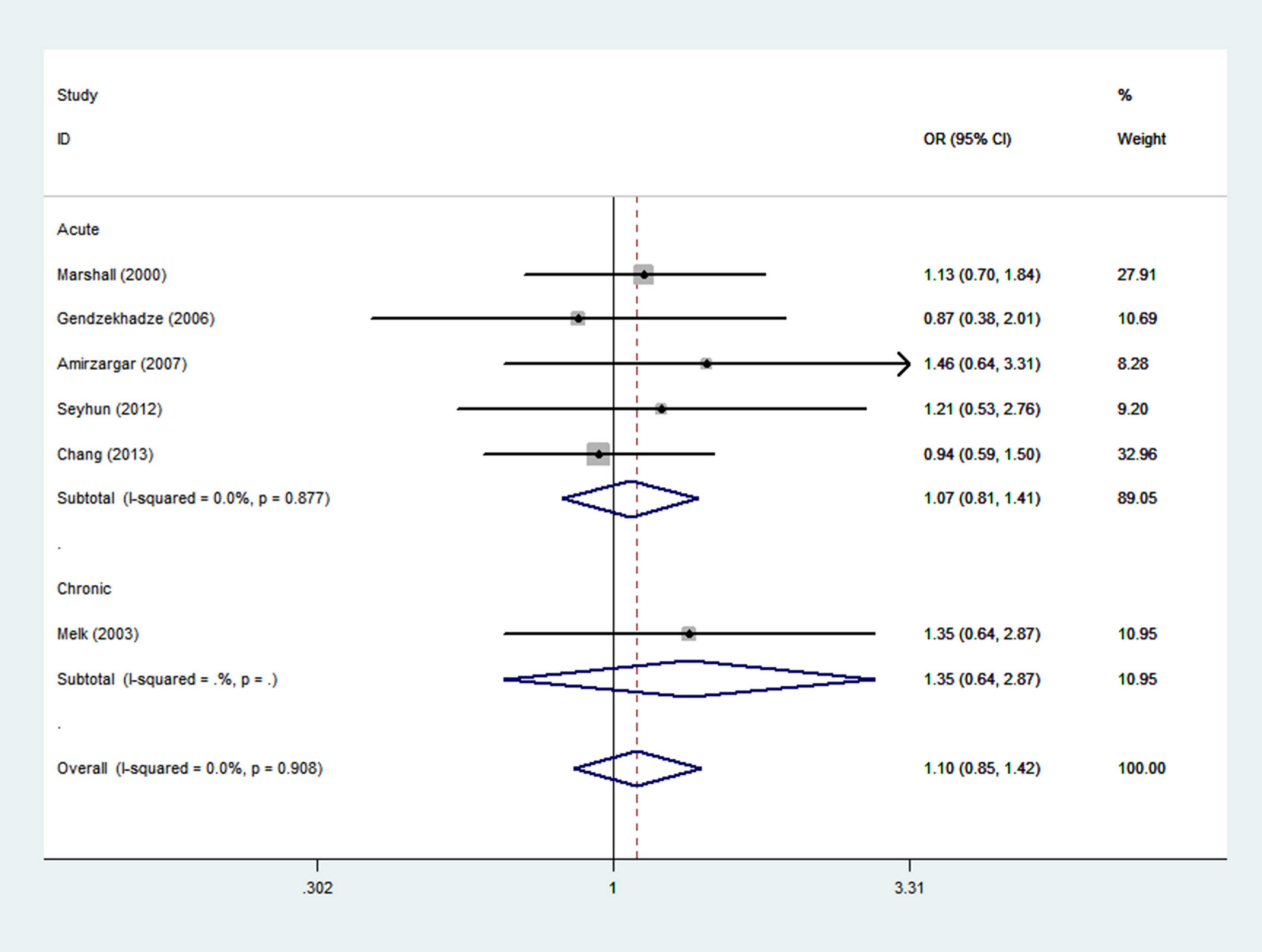
Meta-analysis with a fixed effect model for the association between reject risk and the -592 C/A polymorphism (AC+AA VS CC).

A total of 253 rejection cases and 571 controls in 6 case-control studies were included in the meta-analysis of the relationship between the IL -592 C/A polymorphism and the risk of graft rejection. There were 5 studies that evaluated acute rejection and 1 study that evaluated chronic graft rejection. By ethnic stratification, 3 studies were performed in Europe, 2 studies were performed in Asia, and 1 study was performed with mix ethnic population. We chose the fixed-effects model to synthesize the data from the allelic model, dominant model, additive model and recessive model. Overall, no significant association was found between the -592 C/A polymorphism and graft rejection risk (OR = 1.10, 95%CI, 0.85–1.42, P = 0.47 for AC+AA vs. CC model, Fig 4 and Table 2). In the subgroup analysis based on ethnic and rejection type, we obtained similar results. There was no significant association between the -592 C/A polymorphism and graft rejection risk for acute rejection (OR = 1.07, 95% CI, 0.81–1.41, P = 0.64, Fig 4 and Table 2), chronic rejection (OR = 1.35, 95% CI, 0.64–2.87, P = 0.43, Fig 4 and Table 2), the European population (OR = 1.20, 95%CI, 0.83–1.73, P = 0.33, S3 Fig and Table 2) and the Asian population (OR = 1.05, 95% CI, 0.70–1.60, P = 0.83, S3 Fig and Table 2). The main results of the meta-analysis are shown in Table 2.

### Meta-analysis of the IL-10(-1082,-819,-592) polymorphisms and graft rejection risk

**Fig 5:**
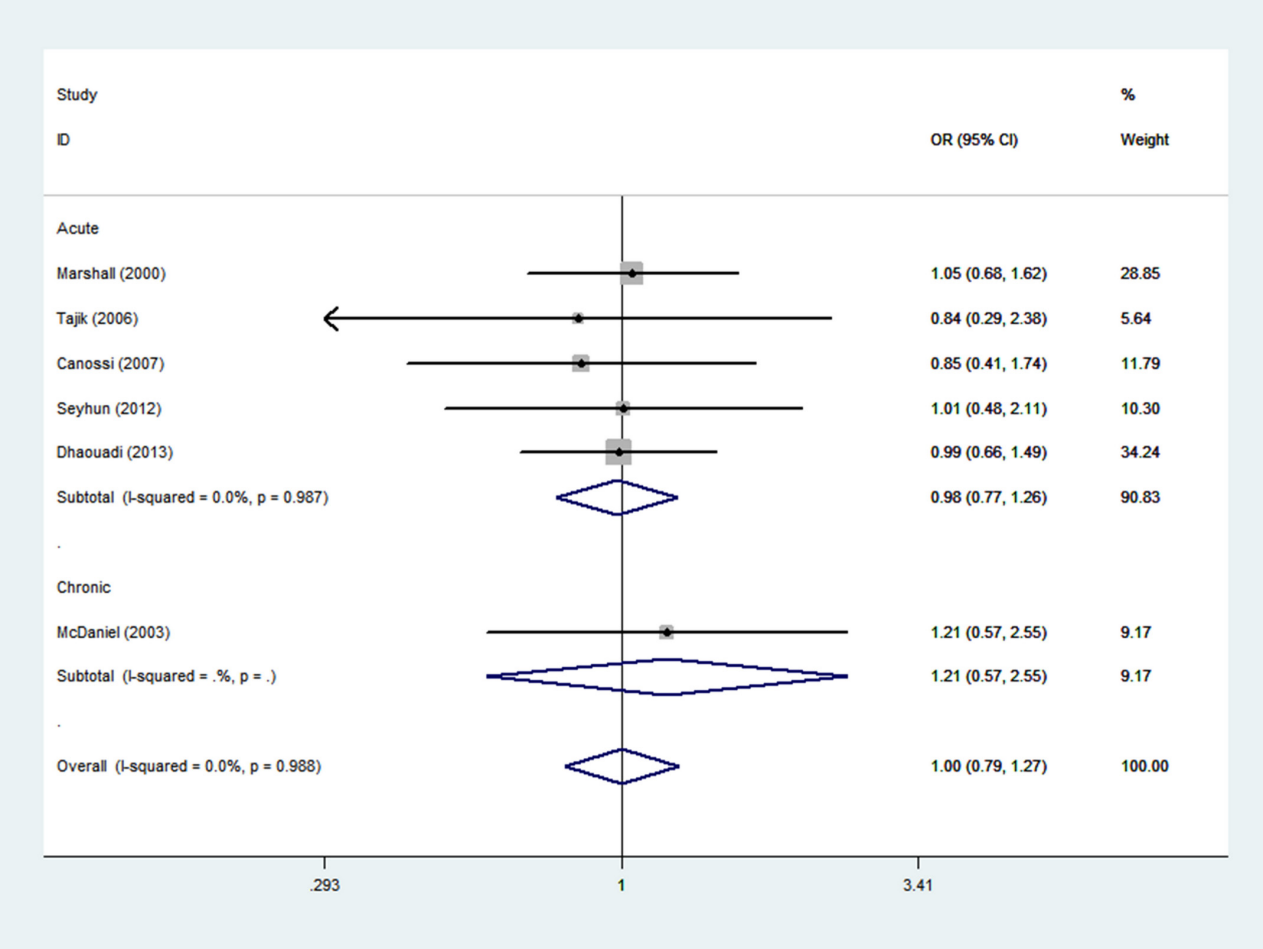
Meta-analysis with a fixed effect model for the association between reject risk and the IL-10 polymorphism (I+L VS H).

A total of 278 rejection cases and 450 controls in 6 case-control studies were included in the meta-analysis of the relationship between the IL-10 (-1082,-819,-592) polymorphisms and the risk of graft rejection. Five studies were performed on acute rejection and 1 was performed on chronic graft rejection. Among them, 3 were from European populations, 1 was from Asia, and 2 were performed with other ethnic populations. The high/intermediate/low expression of the IL-10(-1082, -819, -592) was according to the level of production based on the references [21, 26], the IL-10 GCC/GCC polymorphism was defined as high expression (H), the IL-10 GCC/ACC and GCC/ATA polymorphisms were defined as intermediate producers (I), and the IL-10 ACC, ATA/ACC, and ATA polymorphisms were low producers (L). We conducted I vs. H, L vs. H, I+L vs. H and I vs. L+H models to evaluate the association between the IL-10 (-1082,-819,-592) polymorphisms and the rejection graft risk. Overall, there was no statistical evidence of an association between the IL-10 (-1082,-819,-592) polymorphism and overall graft rejection risk (OR = 1.00, 95%CI, 0.79–1.27, P = 0.98 for I+L vs. H model, Fig 5 and Table 2). Moreover, the subgroup analysis showed similar results. A summary of the results of other genetic comparisons is shown in Table 2 and S4 Fig.

### Publication bias

**Fig 6:**
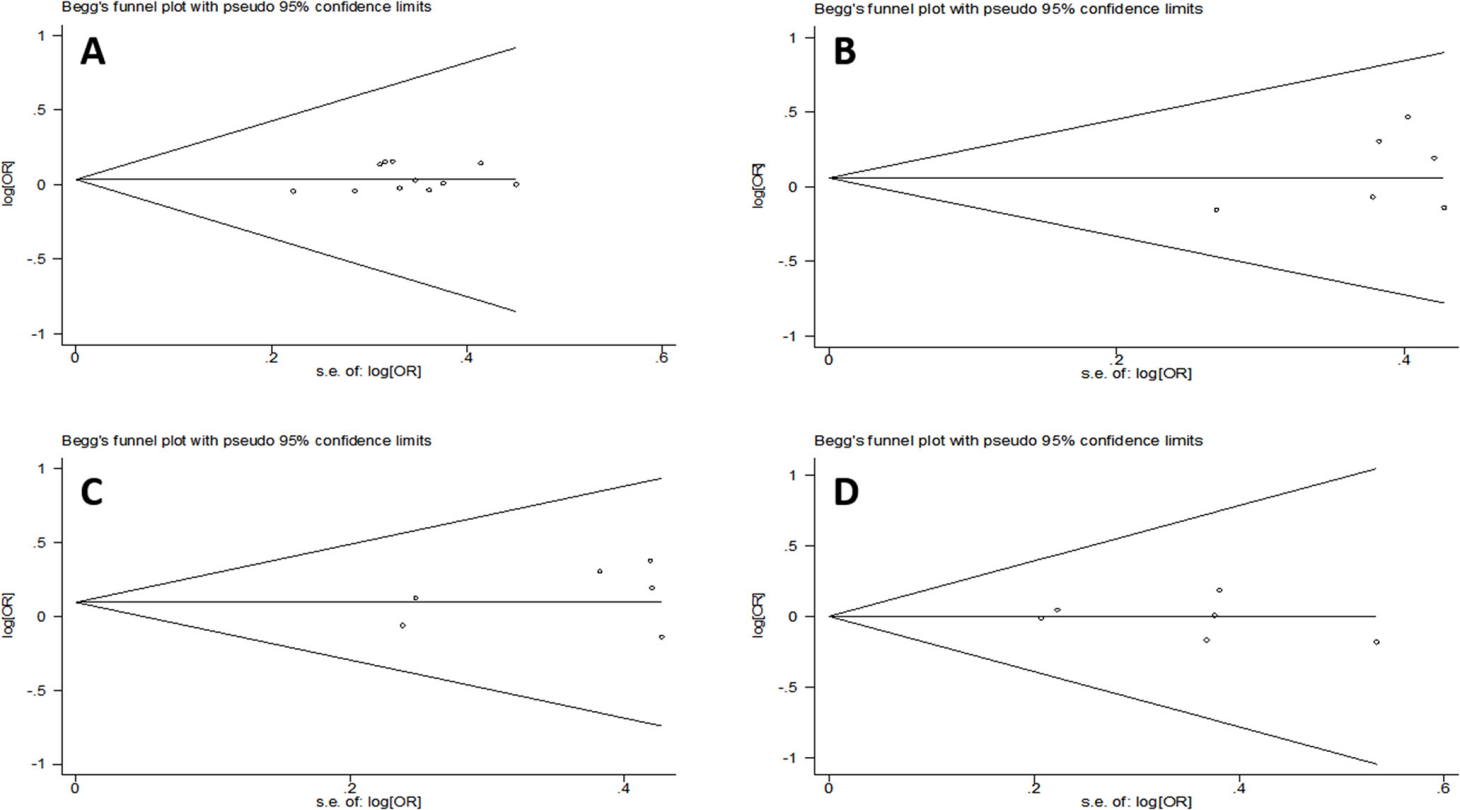
Egger of the published studies bias considered in the meta-analysis. Egger’s regression test showed that P = 0.31 for IL -1082 G/A polymorphism (A), P = 0.25 for IL-819 C/T polymorphism (B), P = 0.37 for IL -592 C/A polymorphism (C), and P = 0.59 for IL-10 (-1082,-819,-592) polymorphism (D), all performed by dominant model.

As reflected by the funnel plots (Fig 6) and the corresponding Egger’s regression test, there was no significant publication bias in all of the pooled studies. The results of the Egger’s regression test were as follows (P = 0.31 for -1082 G/A polymorphism, P = 0.25 for -819 C/T polymorphism, P = 0.37 for -592 C/A polymorphism, and P = 0.59 for IL-10 (-1082,-819,-592) polymorphism, all performed by dominant model).

## Discussion

Allograft rejection is mediated by complex immunologic mechanisms [29]. Cytokines play a very important role in the regulation of cellular activation, differentiation, and function [30]. IL-10 is widely studied among cytokines. It is produced and released from monocytes and lymphocytes [31], has pleiotropic effects in immunoregulation and inflammation and is counted among the anti-inflammatory or Th2 cytokines [32, 33]. Five SNPs tagging the promoter haplotypes of the IL-10 gene have been reported, including SNP-1082, -819 and -592 in the proximal promoter and SNP-3575 and -2763 in the distal promoter [34], but only the three -1082, -819, -592 polymorphisms were most commonly studied in the context of kidney transplantation. It has been demonstrated that the lowest incidence of severe acute graft versus host disease (GVHD) and remission is associated with homozygosity for the IL-10 haplotype, possibly due to the overproduction of IL-10 [35]. When acute rejection or chronic rejection occurred and impaired 1-year graft function in renal allograft recipients, the IL-10 gene expression was significantly elevated [36, 37]. Thus, the IL-10 gene mutation can disrupt the anti-inflammation and immunoregulation function.

The association between the IL-10 gene polymorphism and graft rejection risk has been studied in liver transplantation [38], heart transplantaion [39], and bone marrow transplantation [40], but the association with kidney transplantation has been argued for many years. Sankaran et al. [41] first reported the association between IL-10 gene polymorphism and acute graft rejection following renal transplantation in 1999. They concluded that the IL-10 high producer genotype was significantly associated with multiple rejection episodes in human leukocyte antigen (HLA) mis-matched transplants. Numerous subsequent studies were conducted to confirm the association, but the results were inconclusive. In 2008, a meta-analysis of the association between cytokine gene polymorphisms and outcomes in renal transplantation was performed by Thakkinstian et al. [42] Based on individual data, they suggested a possible association between a 3-SNP-haplotype of IL-10 and poor outcomes in renal transplantation. However, they only included five original studies. It remains unclear whether there is a robust significant association between IL-10 gene polymorphism and graft rejection risk. Therefore, it is necessary to update the estimation of the relationship between this gene variant and graft rejection. We designed this meta-analysis to derive a more precise association between this gene variant and graft rejection risk. In this meta-analysis, we examined the IL-10 gene polymorphism and its relationship with the risk of graft rejection in four genetic models. Overall, no association was found between the IL-10 gene polymorphism and graft rejection risk. It is definite that IL-10 plays an important role of anti-inflammatory and immunomodulatory functions in normal renal physiology, but the results of basic research concerned with the relationship between IL-10 promoter gene polymorphisms and graft rejection are not consistent. Zhang et al. reported that overexpression of IL-10 fails to protect allogeneic islet transplants in no obese diabetic mice [43]. Xu et al. demonstrated a significant association between IL-10 mRNA expression and renal allograft rejection [44]. In addition, kidney is less immunogenic than heart, lung or liver transplantation. It is conceivable that only when the immune system is maximally activated, the impact of some of these gene polymorphisms is manifested. At last, other factors should be considered as well, such as gene-gene interaction or gene-environmental action [14].

In the subgroup analysis based on different types of rejection, we also found there was no association between the IL-10 gene polymorphism and acute graft rejection or chronic graft rejection. Furthermore, considering that the results produced were from different genetic backgrounds, subgroup analyses based on ethnicity were also performed and had similar results. The ethnic populations were mainly from European and Asian countries. Whether other ethnic populations have an association was beyond the explanation of our meta-analysis.

Publication bias did not exist in the overall comparisons, indicating that the results of the present meta-analysis were statistically robust. In addition, subgroup meta-analyses were also performed to determine the heterogeneity of the ethnicity or rejection type, and we found similar results.

For better interpretation of the results, some limitations should be considered in this meta-analysis. We studied the association only in KTx in recipients, while the association between IL-10 polymorphism and the donors was not included. Second, due to a lack of original information for each included subject, we were unable to perform further stratified analyses, such as gender and age. Third, the included studies involved mainly European and Asian populations for subgroup meta-analysis by ethnicity. Data concerning other ethnicities were limited.

In conclusion, the present meta-analysis including 595 rejection patients and 1239 stable graft patients suggested that the IL-10 gene polymorphism was not associated with an increased graft rejection risk. However, due to its limitations, our findings in the present meta-analysis should be confirmed by further chronic rejection and other ethnic population studies.

## Supporting Information

S1 Fig Meta-analysis with a fixed effect model for the association between reject risk and the -1082 G/A polymorphism (GA+AA VS GG). (TIF)

S2 Fig Meta-analysis with a fixed effect model for the association between reject risk and the -819 C/T polymorphism (TA+TT VS CC). (TIF)

S3 Fig Meta-analysis with a fixed effect model for the association between reject risk and the -592 C/A polymorphism (AC+AA VS CC). (TIF)

S4 Fig Meta-analysis with a fixed effect model for the association between reject risk and the IL-10 polymorphism (I+L VS H). (TIF)

S1 File Meta-analysis of genetic association studies checklist. (DOCX)
