# Supplementary figures and images for: Lack of Association between Interleukin-10 Gene Polymorphisms and Graft Rejection Risk in Kidney Transplantation Recipients: A Meta-Analysis
Source: PLoS One. 2015 Jun 2;10(6):e0127540. doi: 10.1371/journal.pone.0127540 (PMC4452718; doi:10.1371/journal.pone.0127540)

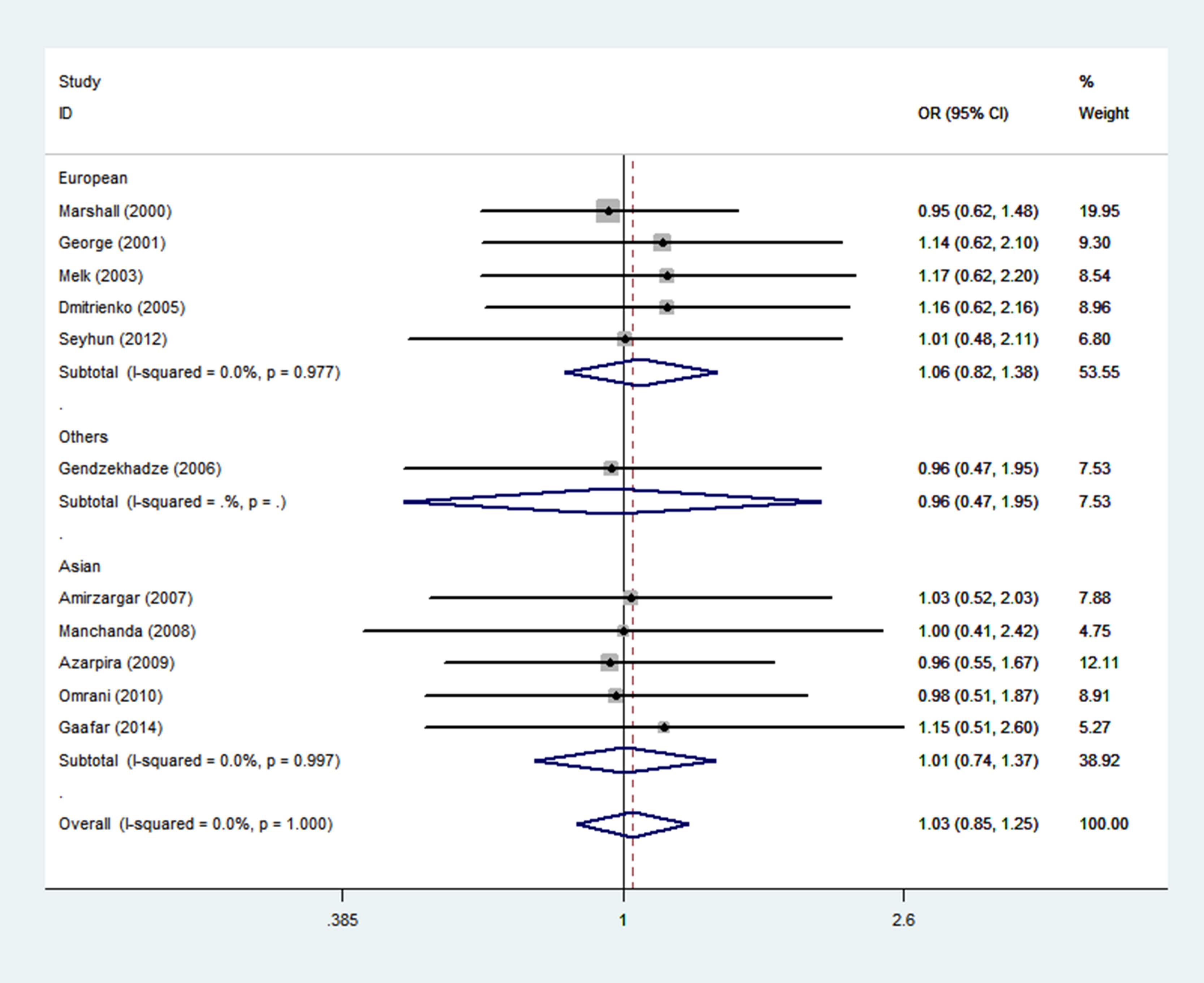

Supplement: S1 Fig — (TIF) [file pone.0127540.s001.tif]

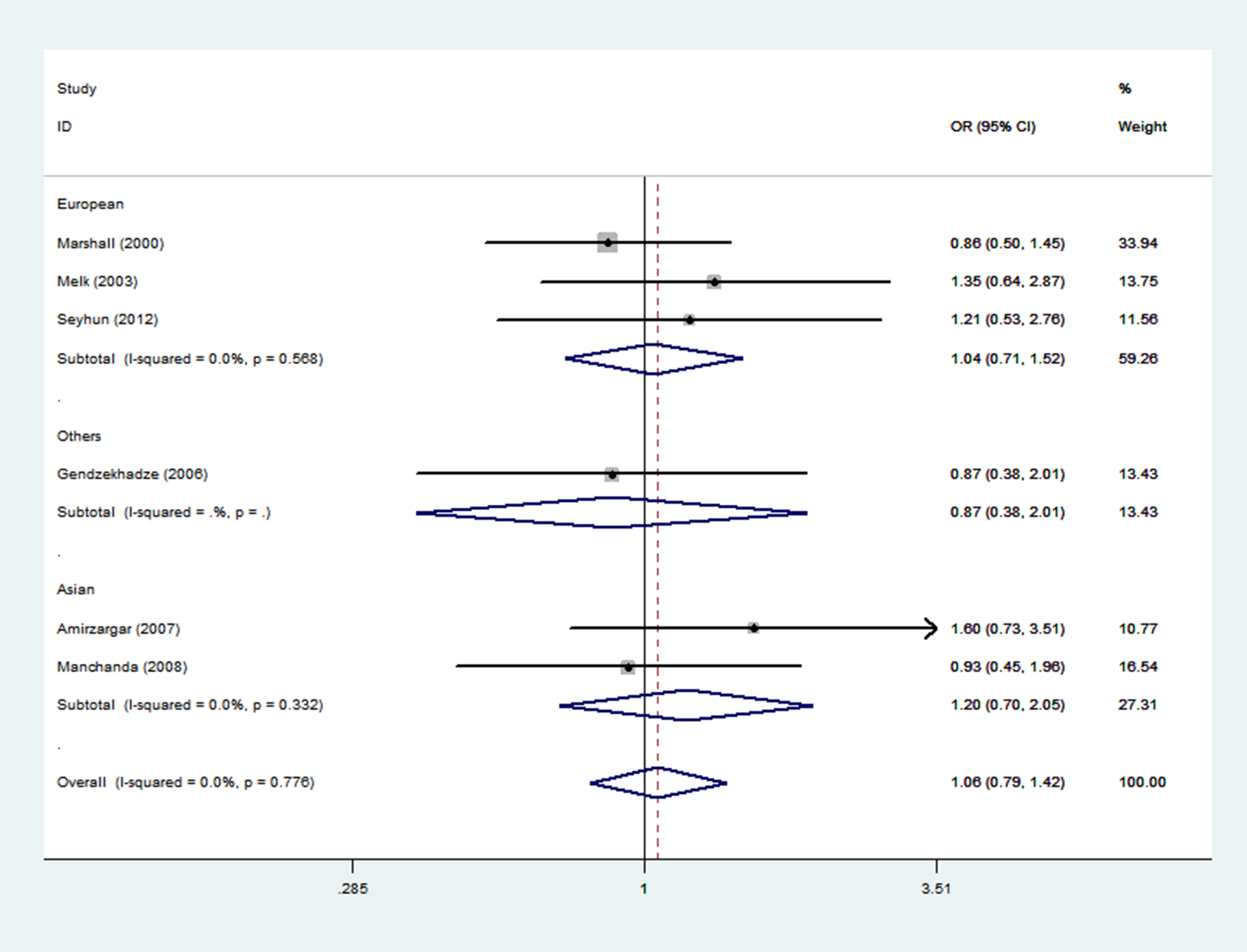

Supplement: S2 Fig — (TIF) [file pone.0127540.s002.tif]

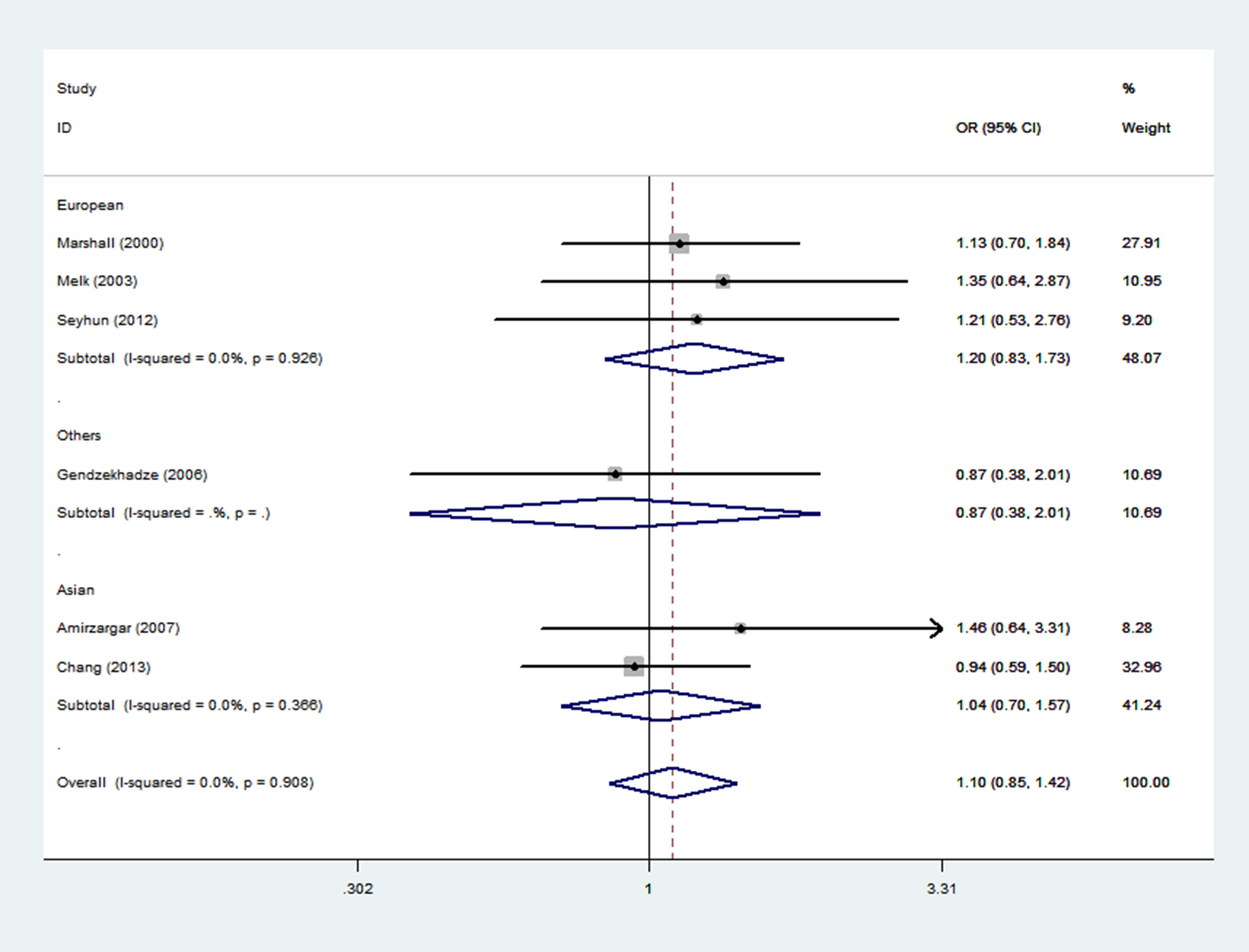

Supplement: S3 Fig — (TIF) [file pone.0127540.s003.tif]

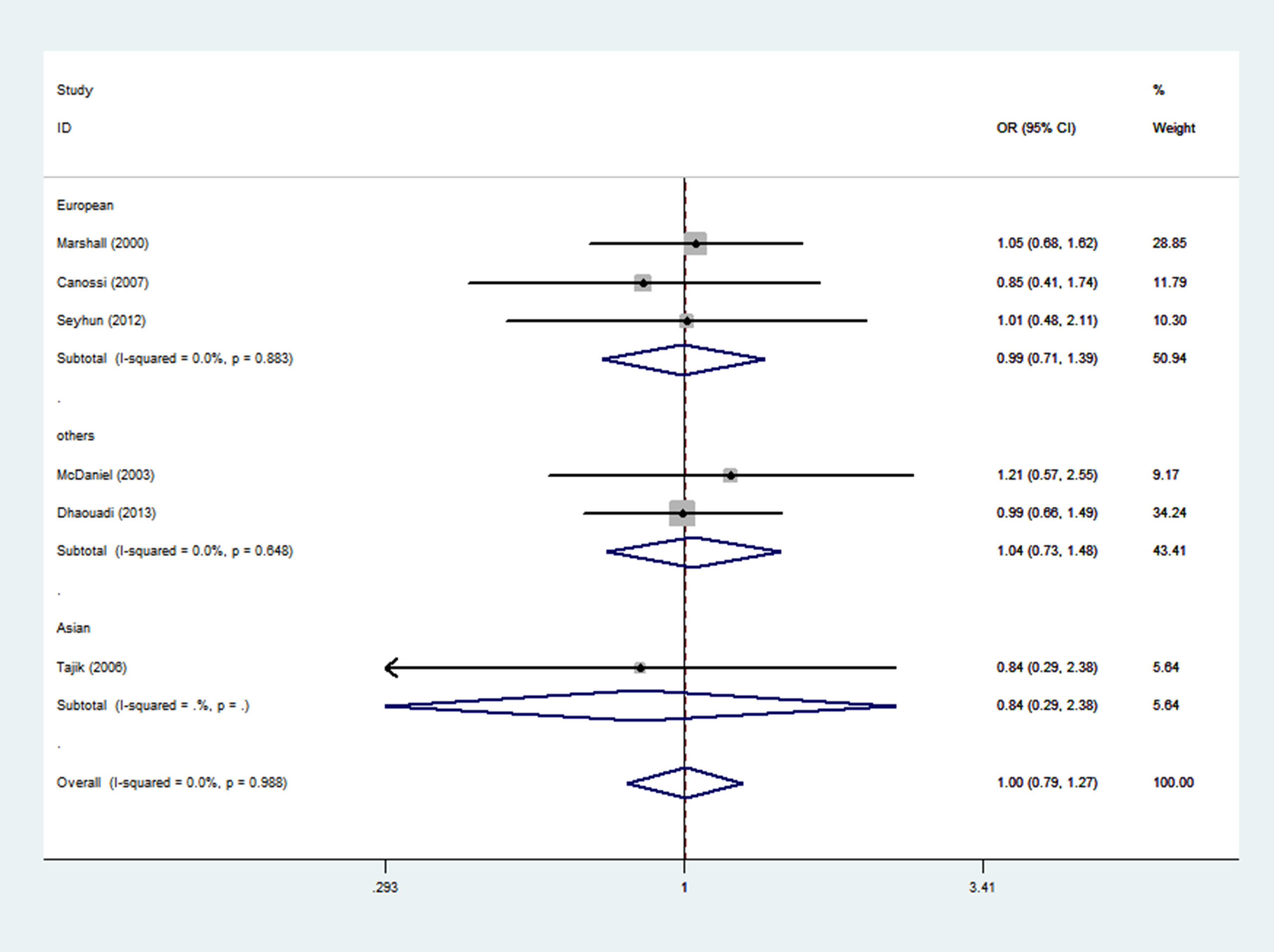

Supplement: S4 Fig — (TIF) [file pone.0127540.s004.tif]
